# Theoretical study on the glycosidic C–C bond cleavage of 3’’-oxo-puerarin

**DOI:** 10.1038/s41598-023-43379-1

**Published:** 2023-09-28

**Authors:** Jongkeun Choi, Yongho Kim, Bekir Engin Eser, Jaehong Han

**Affiliations:** 1 Department of Chemical Engineering, Chungwoon University, 113, Sukgol-ro, Michuhol-gu, Incheon, 22100 Republic of Korea; 2 Department of Applied Chemistry, Institute of Applied Sciences, Kyung Hee University, Yongin, 17104 Republic of Korea; 3 Department of Biological and Chemical Engineering, Aarhus University, Gustav Wieds Vej 10, 8000 Aarhus, Denmark; 4 Metalloenzyme Research Group, Department of Plant Science and Technology, Chung-Ang University, 4726 Seodong-daero, Anseong, 17546 Republic of Korea

**Keywords:** Biocatalysis, Biocatalysis, Biophysical chemistry, Biocatalysis, Computational chemistry

## Abstract

Puerarin, daidzein *C*-glucoside, was known to be biotransformed to daidzein by human intestinal bacteria, which is eventually converted to (*S*)-equol. The metabolic pathway of puerarin to daidzein by DgpABC of *Dorea* sp. PUE strain was reported as puerarin (**1**) → 3’’-oxo-puerarin (**2**) → daidzein (**3**) + hexose enediolone (**C**). The second reaction is the cleavage of the glycosidic C–C bond, supposedly through the quinoid intermediate (**4**). In this work, the glycosidic C–C bond cleavage reaction of 3’’-oxo-puerarin (**2**) was theoretically studied by means of DFT calculation to elucidate chemical reaction mechanism, along with biochemical energetics of puerarin metabolism. It was found that bioenergetics of puerarin metabolism is slightly endergonic by 4.99 kcal/mol, mainly due to the reaction step of hexose enediolone (**C**) to 3’’-oxo-glucose (**A**). The result implied that there could be additional biochemical reactions for the metabolism of hexose enediolone (**C**) to overcome the thermodynamic energy barrier of 4.59 kcal/mol. The computational study focused on the C–C bond cleavage of 3’’-oxo-puerarin (**2**) found that formation of the quinoid intermediate (**4**) was not accessible thermodynamically, rather the reaction was initiated by the deprotonation of 2’’C–H proton of 3’’-oxo-puerarin (**2**). The 2’’C-dehydro-3’’-oxo-puerarin (**2a2C**) anionic species produced hexose enediolone (**C**) and 8-dehydro-daidzein anion (**3a8**), and the latter quickly converted to daidzein through the daidzein anion (**3a7**). Our study also explains why the reverse reaction of *C*-glycoside formation from daidzein (**3**) and hexose enediolone (**C**) is not feasible.

## Introduction

**Figure 1:**
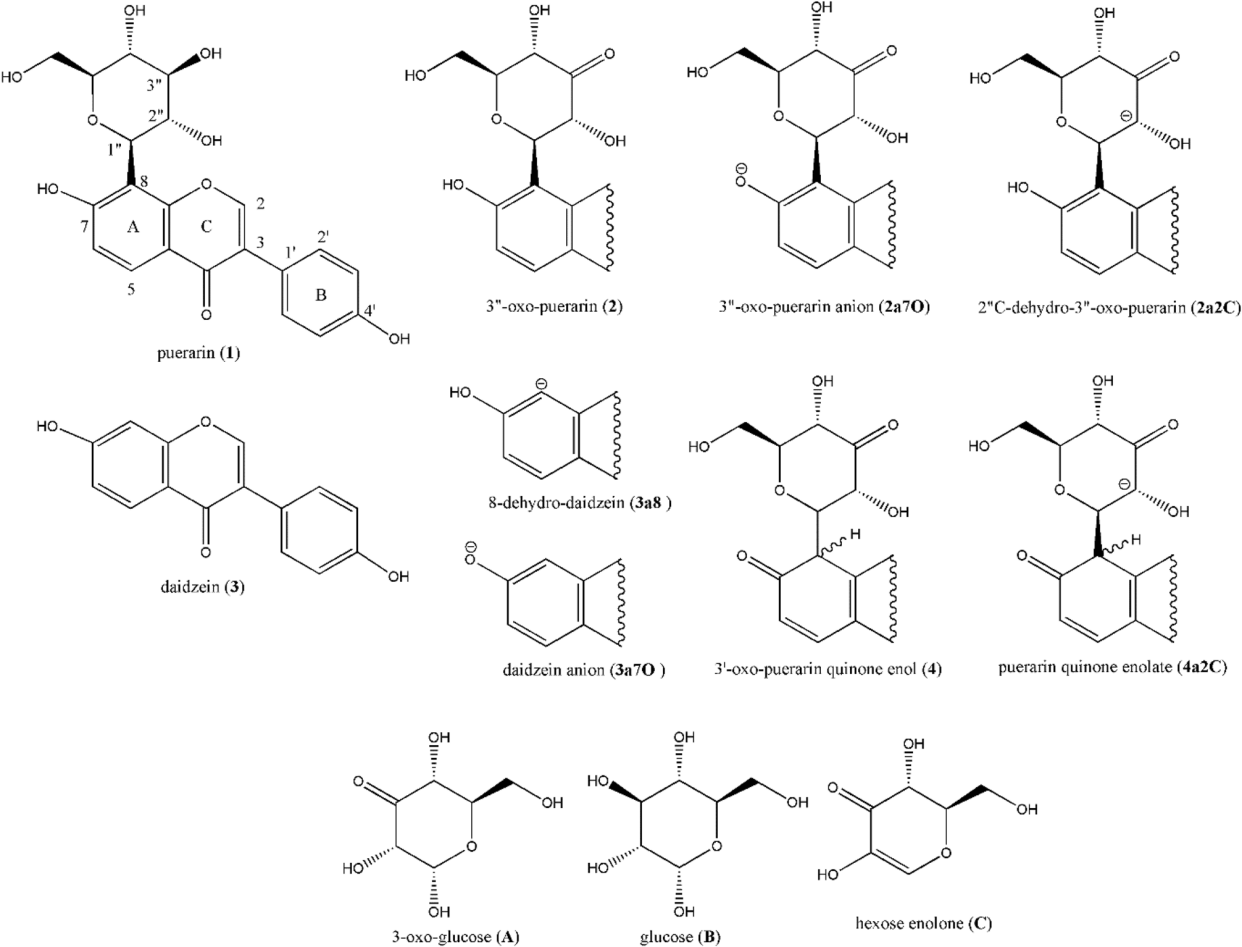
Structures of molecules studied in this work.

Due to the unique chemical conversion of the glycosidic C–C bond cleavage, intestinal metabolism of flavonoid *C*-glycosides has been ever growing research interests^1^. Biological C–C bond cleavage reaction was first known from the deglycosylation of mangiferin by human gut flora^2^. Especially, biotransformation of puerarin (**1**) to daidzein (**3**) has been extensively investigated^3–5^, to complete the (*S*)-equol producing metabolic pathway in human gut (Fig. 1)^6–8^ After reporting the puerarin-metabolizing gut bacteria^3^, we have been studying the chemical reaction mechanism of biochemical C–C and C–O bond cleavage reactions operated by human gut bacteria to investigate novel enzyme reaction systems^9–11^. Whereas biosynthesis of flavonoid *C*-glycosides appears to follow the same mechanism as the biosynthesis of flavonoid *O*-glycosides by utilizing UDP-glucose as glucose donor^12^, catabolism of flavonoid *C*-glycoside involving glycosidic C–C bond cleavage looks very different from the hydrolysis of flavonoid *O*-glycosides. Especially, the reaction mechanism has been scarcely studied at the molecular level.

**Figure 2:**
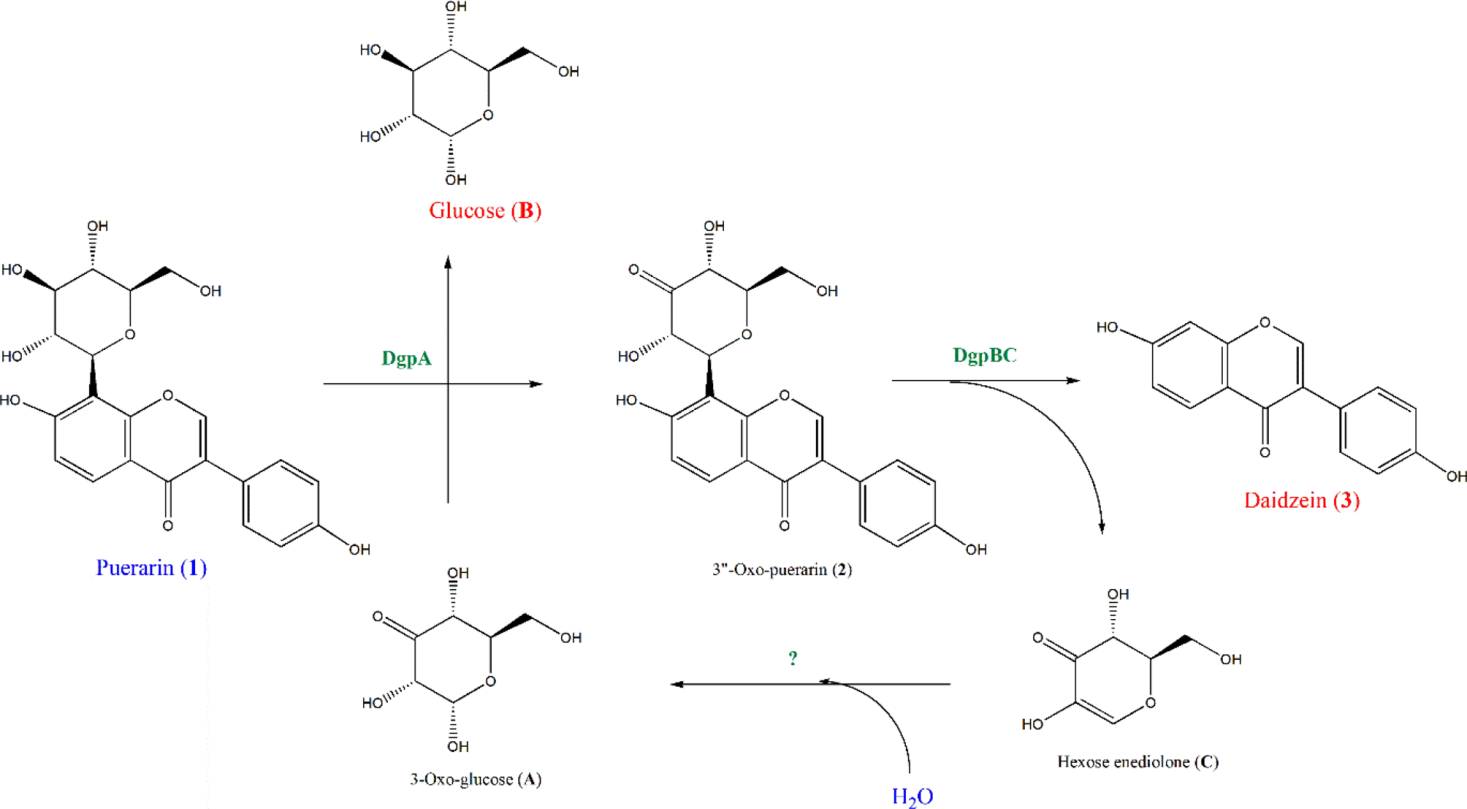
Puerarin (**1**) metabolism by human gut bacterium, *Dorea* sp. PUE. Puerarin (**1**) is oxidized to 3’’-oxo-puerarin (**2**) with the reduction of 3-oxo-glucose (**A**) to glucose (**B**) by DgpA. 3’’-Oxo-puerarin (**2**) is then converted to daidzein (**3**) and hexose enediolone (**C**) by DgpBC. The enzyme involved in the stereoselective conversion of hexose enediolone (**C**) to 3-oxo-glucose (**A**) was not identified yet.

Recently, the enzyme systems of deglycosylation of *C*-glycosides were identified. It was also reported that the actual substrate of puerarin *C*-deglycosidase was 3’’-oxo-puerarin (**2**), rather than puerarin (**1**)^13,14^. The hexose enediolone metabolite (**C**) and daidzein (**3**) were also identified as products of 3’’-oxo-puerarin (**2**) metabolism by the over-expressed enzyme system^15^. Therefore, metabolic pathway of puerarin (**1**) has been established now as shown in Fig. 2. It is noteworthy that the direct glycosidic C–C bond cleavage of flavonoid *C*-glucosides has never been reported so far. It appears the activation of *C*-glycoside glucose moiety, such as oxidation of C3’’-carbon, is necessary for the cleavage of the glycosidic C–C bond cleavage. Similar biochemical mechanism has been reported from the glycosidic C–O bond cleavage by glycoside hydrolase family 4 and 109 β*-*glucosidases^16,17^.

**Figure 3:**
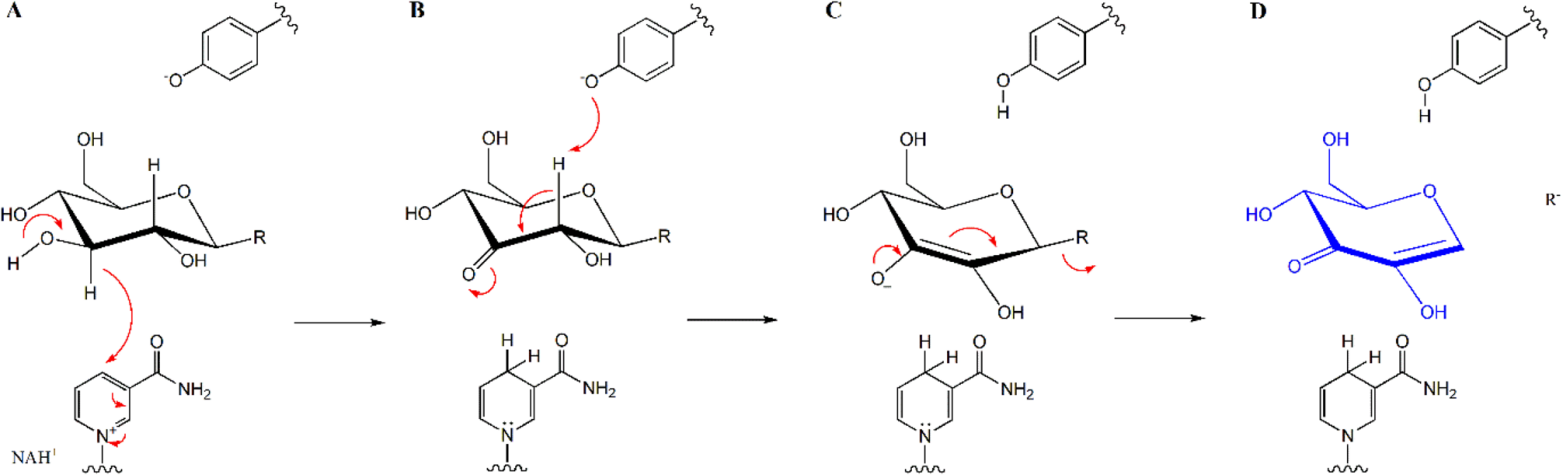
Possible metabolism of puerarin (**1**) by the mechanism of GH4 and 109 enzymes. The carbanion species, its endiolate tautomer is shown at (**C**), is the key intermediate in the mechanism. In the case of puerarin (**1**) metabolism, DgpA produces 3-oxo-puerarin (**2**) (**A,B**) and the next reactions (**B,D)** are catalyzed by DgpBC to produce hexose enediolone (blue in (**D**)) and aglycone. R in the structures may be *O*-, *C*-, or *N*-glycosides.

Glycoside metabolism through 3-keto intermediate formation seems to be quite common in nature^18^. Various *C*- and *O*-glycosides, including flavonoids, ginsenosides, and anthraquinones are metabolized by NAD^+^/FAD-utilizing enzymes^16–19^. DgpA converting puerarin (**1**) to 3’’-oxo-puerarin (**2**) also belongs to the same enzyme family, GH 109, of which the mechanism was established (Fig. 3)^17,20^. The common mechanism of GH 4 and 109 enzymes employs the 2’’C-carbanion intermediate. However, the reaction of DgpA is unique in that the enzyme does not regenerate NAD^+^ by the known mechanism, instead NAD^+^ is recovered from the reduction of 3-oxo-glucose^15^. If DgpA metabolizes puerarin (**1**) according to the mechanism of the same family enzyme, puerarin (**1**) could be hydrolyzed to daidzein (**3**) and glucose (**B**) through the 3’’-oxo intermediate (**2**)^17^. But, DgpA stops the reaction at the stage of **B** in Fig. 3 for unknown reasons. The next C–C bond cleavage reaction (**C** in Fig. 3) is catalyzed by DgpBC. We suspect that the stability of the carbanion intermediate requires DgpBC for the next step of C–C bond cleavage reaction.

**Figure 4:**
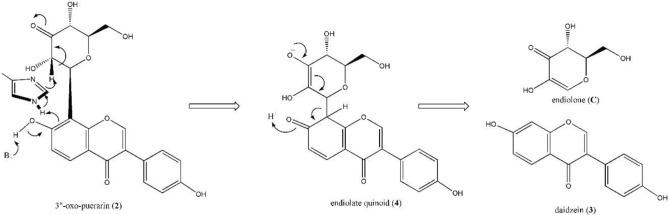
Proposed mechanism of biochemical C–C bond cleavage by PUE strain. Metal aqua species (designated as B) acts as Lewis base to initiate the reaction, and the His residue plays a key role in the dearomatization of A-ring and deprotonation of C2’’-H of 3’’-oxo-puerarin (**2**) at the same time. The key enolate quinoid intermediate (**4**) proceeds the β-elimination (reverse reaction of Michael addition) type C–C bond cleavage.

Based on the docking model and biochemical studies^21^, the reaction mechanism of the C–C bond cleavage by puerarin *C*-deglycosidase (DgpBC) was proposed recently as shown in Fig. 4. The proposed mechanism requires a divalent metal ion and histidine residue in the active site for the enzyme activity. Especially, the metal aqua species was claimed to act as a Lewis base to form the unusual quinoid intermediate (**4**). However, the divalent metal aqua species should act as a Lewis acid or a strong electrophile rather than a Lewis base^22,23^. Furthermore, we judge breaking aromaticity of flavonoid A-ring may demand practically inaccessible activation energy. For example, it is known that resonance stabilization energy of benzene is about 36 kcal/mol.

Alternatively, C–C bond cleavage of 3’’-oxo-puerarin (**2**) could adopt the reaction mechanism of GH 109, as shown in the reaction step **C** to **D** in Fig. 3. The reaction step is simple and produces hexose endiolone (**C**) and daidzein (**3**), as observed experimentally. Especially, the formation of carbanion species (**2a2C**) is precedented (Fig. 3)^17^. However, the elucidation of the actual reaction mechanism would require extensive investigation, because breaking C–C bond of 3’’-oxo-carbanion glycosides could be different from the cleavage of C–O bond of the 3’’-oxo-carbanoin species.

In this study, biochemical energetics of the newly discovered intestinal puerarin (**1**) metabolism (Fig. 2), as well as the chemical reaction mechanism of C–C bond cleavage of 3’’-oxo puerarin (**2**), was investigated by computational chemistry. Especially, accessibility of the quinone intermediate (**4**) formation as the initiation of glycosidic C–C bond cleavage reaction was evaluated (Fig. 4). Finally, we have proposed a new chemical reaction mechanism of the C–C bond cleavage reaction of 3’’-oxo-puerarin (**2**).

## Results and discussion

### Conformational analysis

**Figure 5:**
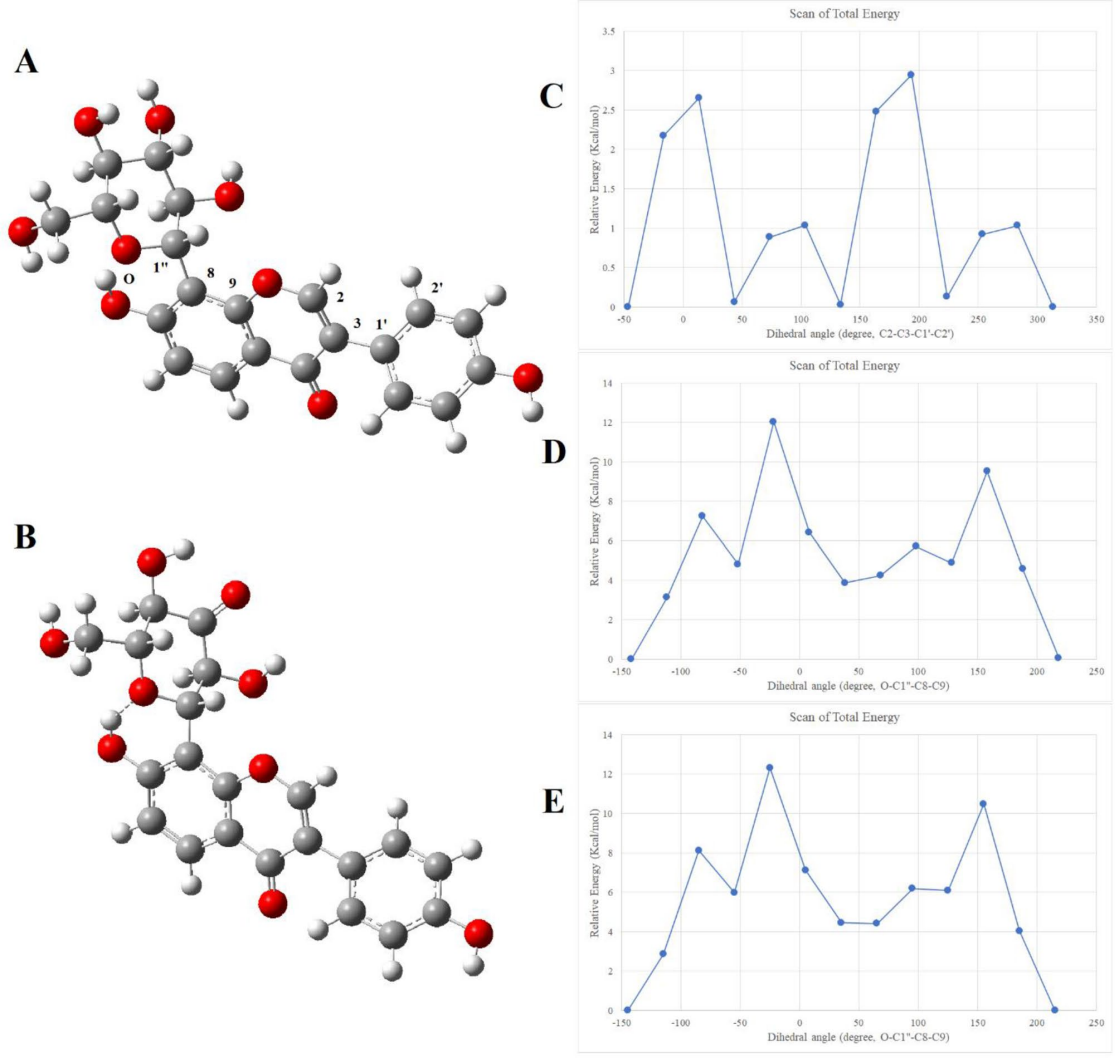
Rotational energy changes of puerarin (**1**) and 3’’-oxo-puerarin (**2**). Stable conformer of puerarin (**A**) and 3’’-oxo-puerarin (**B**). Both were stabilized by H-bonding interactions between glucopyranosyl oxygen and 7O–H. Four stable conformers were found from B-ring rotamers (**C**). For the glycosyl rotamers of **1** and **2**, only one stable conformer was found for each ((**D,E**), respectively)). H-bonding interaction was emphasized by dashed line in (**B**).

The rotational energy barriers of puerarin B-ring and glucopyranosyl group of puerarin (**1**) and 3’’-oxo-puerarin (**2**) were calculated by DFT calculations using the relaxed scan. It was found that there were four stable B-ring rotamers within 3 kcal/mol of energy barrier (Fig. 5C). The conformation of 4’O–H dihedral angle was not investigated, because the energy barrier is negligible. Likewise, conformation of B-ring would not be important for the cleavage of C–C bond cleavage because B-ring is far from the reaction site.

The conformational analysis of the glucopyranosyl group of puerarin (**1**) and 3’’-oxo-puerarin (**2**) resulted in a few interesting points. First, the rotational energy barriers for both compounds were found about 12 kcal/mol (Fig. 5D, E). There was only one stable conformer for both compounds (Fig. 5A, B), and the distribution of each conformer was more than 99.99% from Boltzmann equation. It was clear from the optimized structures that the stable conformers utilized H-bonding interaction between 7O-H group and glucopyranosyl oxygen atom. The distances of 1.825 Å and 1.813 Å were found for **1** and **2**, respectively.

The geometry-optimized structure of 3’’-oxo-puerarin (**2**) shown in Fig. 5B and the high rotational energy barrier found from the 3’’-oxo-glycosyl group conformers of **2** (Fig. 5E) are significant in the theoretical study of the reaction mechanism, because the active conformation of **2** proposed in Fig. 4 is not possible. In other words, deprotonation of 7O–H could be achieved by the protonation of 8C aromatic carbon, but which cannot be directly related to the deprotonation of 2’’C–H as previously suggested^21^, due to the spatial position of 2’’C–H.

### Biochemical energetics of puerarin (*1*) metabolism

In the net reaction, puerarin (**1**) can be considered to be metabolized to daidzein (**3**) and glucose (**B**) by addition of water (Eq. 4). However, biochemical *C*-deglycosylation reaction of puerarin (**1**) is known to begin with the regioselective oxidation of 3’’-hydroxyl group of puerarin (**1**) to 3’’-oxo-puerarin (**2**) by DgpA. Then, the glycosyl unit of **2** is converted to hexose enediolone (**C**) by DgpBC. The product **C** appears to be converted to glucose (**B**) in the later biochemical reaction (Fig. 2). The first reaction of puerarin (**1**) metabolism is catalyzed by DgpA of *Dorea* sp. PUE strain through the NAD^+^-mediated regioselective oxidation of glycosyl group of puerarin (**1**), which is also coupled to the reduction of 3-oxo-glucose^15^. After DFT calculations of the free energy (Table S1), we have found that the Gibbs free energy values between puerarin (**1**) + 3-oxo-glucose (**A**) and 3’’-oxo-puerarin (**2**) + glucose (**B**) in Eq. (1) were almost same in ethanol, so that the reaction is almost at equilibrium. It was slightly endergonic by 0.18 kcal/mol in ethanol and slightly exergonic by 0.65 kcal/mol in *n*-octanol (Eq. 1).

The free energy change for the C–C bond cleavage reaction of 3’’-oxo-puerarin (**2**), catalyzed by DgpBC of *Dorea* sp. PUE was found also slightly endergonic by 0.22 kcal/mol in ethanol and 0.64 kcal/mol in *n*-octanol, respectively. The reaction mechanism of Eq. (2) is of a great interest in this research, and the reaction appeared reversible based on thermodynamic data with the equilibrium constant of 0.69 and 0.34 in ethanol and *n*-octanol, respectively, at room temperature.

The last reaction of puerarin metabolism is the hydration of hexose enediolone (**C**) to form 3-oxo-glucose (**A**), but the biochemical reaction has not been elucidated yet. Formation of 3-oxo-glucose (**A**) was found to be endergonic by 4.59 kcal/mol (Eq. 3). The Gibbs free energy change of the last reaction, Eq. (3), strongly suggested that it may not be a single reaction step catalyzed by a single gene product. In fact, eight genes of DgpA-H were reported from *dgp* operon^13^. ATP-driven reaction of hexose enediolone (**C**) metabolism may be possible, too.

1 $${\text{puerarin }}\left( {\mathbf{1}} \right) \, + 3{\text{-oxo-glucose }}\left( {\mathbf{A}} \right) \to {3}{\text '} {\text '}{\text{-oxo-puerarin }}\left( {\mathbf{2}} \right) \, + {\text{ glucose }}\left( {\mathbf{B}} \right) \, \quad + 0.18\;{\text{kcal}}/{\text{mol}}$$

2 $${3}{\text '} {\text '}{\text{-oxo-puerarin }}\left( {\mathbf{2}} \right) \to {\text{daidzein }}\left( {\mathbf{3}} \right) \, + {\text{ hexose enediolone }}\left( {\mathbf{C}} \right) \quad + 0.22\;{\text{kcal}}/{\text{mol}}$$

3 $${\text{hexose enediolone }}\left( {\mathbf{C}} \right) \, + {\text{H}}_{{2}} {\text{O}} \to 3{\text{-oxo-glucose }}\left( {\mathbf{A}} \right)\quad + {4}.59\;{\text{kcal}}/{\text{mol}}$$

4 $${\text{puerarin }}\left( {\mathbf{1}} \right) \, + {\text{ H}}_{{2}} {\text{O}} \to {\text{daidzein }}\left( {\mathbf{3}} \right) \, + {\text{ glucose }}\left( {\mathbf{B}} \right) \quad + {4}.99\;{\text{kcal}}/{\text{mol}}$$

From the biochemical energetics, the net reaction of puerarin metabolism was endergonic by 4.99 kcal/mol (Eq. 4). Biochemical energetics of puerarin (**1**) metabolism to daidzein (**3**) and glucose (**B**) was found to be thermodynamically unfavorable at the standard conditions. This may be the one of the reasons why DgpA alone cannot cleave the C–C bond.

### Mechanism of C–C bond cleavage

It was speculated that the Mn^2+^ ion in the active site of *C*-deglycosidase had a specific catalytic function during the enzyme reaction^24^. However, the metal ion coordination environment of the protein X-ray crystallographic structures of DgpBC was found very diverse, and distorted octahedral (PDB: 7bvr, 7exb, 7exz), distorted trigonal pyramidal (PDB: 7bvs), and square pyramidal (PDB: 7xrf) geometries were identified^21,25^. In fact, it was reported that the substitution of the Mn^+2^ ion to other divalent transition metal ions had similar effects on the enzyme activity^21^. Therefore, it is believed that any divalent metal ions are sufficient for the catalytic activity of DgpBC in the active site. At present, it is too early to tell whether DgpBC is a Mn-dependent enzyme or the divalent metal ion is directly involved in the C–C bond cleavage.

The previous proposed mechanism for the C–C bond cleavage initiates the reaction with deprotonation of 7-OH group of 3’’-oxo-puerarin (**2**), which is claimed to be facilitated by Mn-OH species (Fig. 3). However, the basicity of the Mn-aqua species is expected to be very weak and rather it should be considered as Lewis acid, as mentioned above. Although the unidentified metal ion may have a function during the catalysis, theoretical glycosidic C–C bond cleavage reaction focused on the 3’’-oxo-puerarin (**2**) substrate without intervention by metal ion was investigated in this study. Furthermore, it is practically impossible to investigate all the possible metal ions, as well as including all the possible spin states of each metal ion, in the computational study.

The acidity of phenolic hydroxyl group has been studied^26^, and the 7-OH group of isoflavone was known to be easily deprotonated in the aqueous solution^27^. In this work, we have shown that deprotonation of puerarin (**1**) and 3’’-oxo-puerarin (**2**) 7-OH proton would be much more difficult than the other isoflavones due to the H-bonding with the glucopyranosyl oxygen atom. Especially, breaking the H-bonding interaction in **2** would require a significant amount of energy in the active site of DgpBC, compared to the aqueous medium. Therefore, the formation of 3’’-oxo-puerarin anionic species, **2a7O**, would be strenuous for the next reaction, such as quinoid anionic species formation.

**Figure 6:**
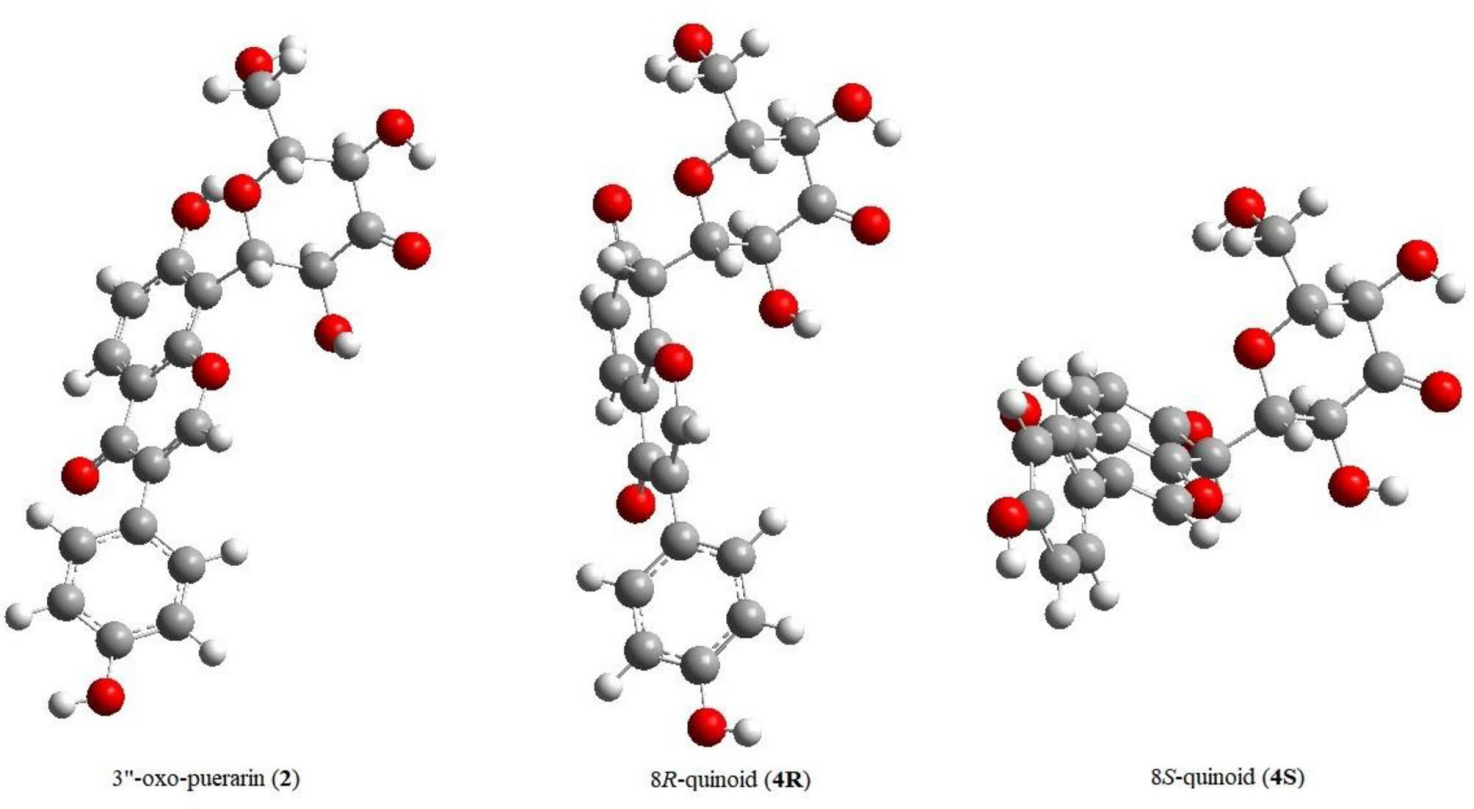
Structures of 3’’-oxo-puerarin (**2**) and 3’’-oxo-puerarin quinoid diastereomers (**4**).

If the quinoid intermediate (**4**) formed as suggested in Fig. 3, it would generate two stereoisomers at the C8 position. The geometry optimization results of two diastereomers of the quinoid intermediate (**4R** and **4S**) found significantly different molecular structures (Fig. 6). Considering most specific substrate interactions in the active site are mainly around the glycosyl unit, the formation of (8*S*)-quinoid (**4S**) may not be favored, due to the drastic structural changes of the aglycone moiety from 3’’-oxo-puerarin (**2**). When the Gibbs free energy was compared between (**2**) and (**4R**), the quinoid species **4R** was unstable compared to 3’’-oxo-puerarin (**2**) by 17.12 kcal/mol. Though, it should be pointed out that the 2’’C–H deprotonated species of **4R**, **4a2C** in Fig. 1, was quickly converted to the products of hexose enediolone (**C**) and daidzein anion (**3a7O**), upon geometry optimization.

To evaluate the feasibility of the quinoid intermediate (**4**) formation, imidazole was introduced above the *si*-face of **2a7O** and the geometry was optimized. With the geometry optimized structure, the proton of imidazole 1N-H was moved to the 8C center of **2a7O** by using relaxed scan method. It produced imidazole anion and with the quinoid intermediate (**4**), but the potential energy increased by more than 42 kcal/mol. Even though the quinoid intermediate (**4**) could facilitate the cleavage of C–C bond to form the products, breaking aromaticity of flavonoid A-ring to form the quinoid intermediate was found thermodynamically disapproved due to the high energy barrier. Therefore, we rejected the quinoid species as an intermediate of the reaction and searched the other deprotonated species of 3’’-oxo-puerarin (**2**), to test whether it is feasible to cleave the C–C bond without formation of quinoid intermediates.

### A new mechanism of biochemical C–C bond cleavage

Once 3’’-oxo-puerarin (**2**) binds in the active site of puerarin *C*-deglycosidase, the first step of catalysis would be deprotonation of 3’’-oxo-puerarin (**2**). Because the formation of 3’’-oxo-puerarin (**2**) from DgpA reaction makes 2’’C–H acidic^15^, deprotonation of 2’’C–H was chosen as an initiation of the glycosidic C–C bond cleavage reaction. The same deprotonation of the acidic 2’’C–H was experimentally proved by the deuterium incorporation at 2’’C and kinetic isotope effect measurement from GH109 family enzyme^17^. When two possible anionic species of 3’’-oxo-puerarin (**2**) were compared for their stability, the Gibbs free energy of 2’’C-dehydro-3’’-oxo-puerarin (**2a2C**) anionic species was higher than that of 3’’-oxo-puerarin anion (**2a7O**) by 12.17 kcal/mol in *n*-octanol, mainly because of the H-bonding interaction mentioned above. Accordingly, we thought **2a7O** was too stable to proceed to the next reaction step. Besides, the formation of **2a2c** in the active site was supported from the identification of 2’’-deuterio-3’’-oxo-puerarin (2) in the D_2_O-substituted buffer in the presence of DgpA^24^.

**Figure 7:**
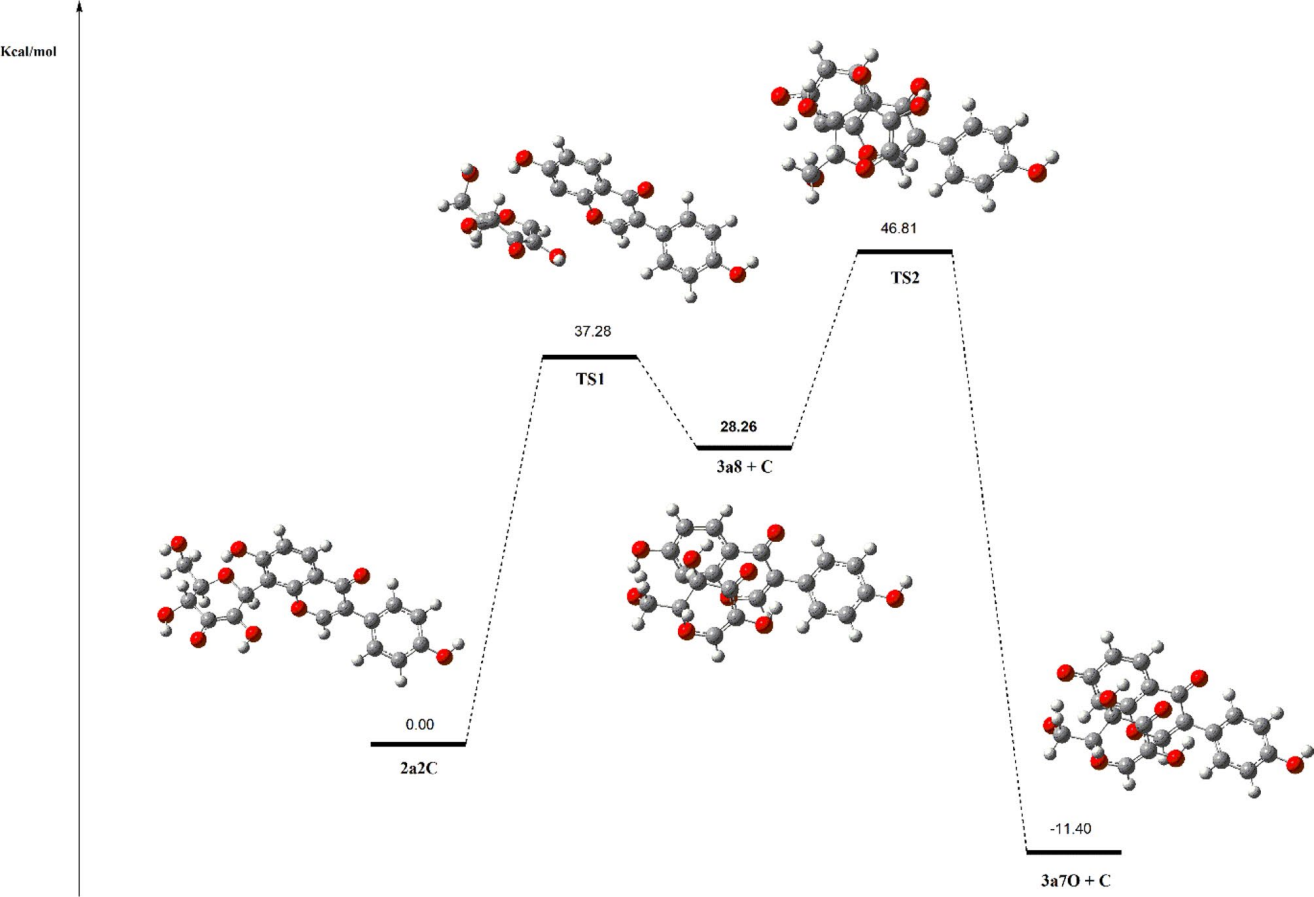
The proposed reaction mechanism of C–C bond cleavage of 3’’-oxo-puerarin (**2**). The initiation of catalysis begins with 2’’-CH deprotonation, Direct cleavage of C1’’–C8 bond forms hexose enediolone (**C**) and 8-dehydro-daidzein (**3a8**). The 8-dehydro-daidzein (**3a8**) is converted to 7-OH deprotonated daidzein **3a7** by intramolecular proton transfer.

From the 2’’C-dehydro-3’’-oxo-puerarin anionic species (**2a2C**), we proposed direct glycosidic C–C bond cleavage of **2a2C** by Michael-type β-elimination, resulting in the C8–C1’’ bond cleavage products of hexose enediolone (**C**) and 8-dehydro-daidzein (**3a8**). However, the **C** + **3a8** product was found to be more unstable than **2a2C** by 28.26 kcal/mol. It was mainly due to the instability of 8-dehydro-daidzein (**3a8**) in the cleavage products, as found from the free energy comparison between daidzein anion (**3a7**) and 8-dehydro-daidzein (**3a8**). Daidzein anion (**3a7**) was more stable than 8-dehydro-daidzein (**3a8**) by 25.14 kcal/mol. Therefore, we suggested **3a8** as a reaction intermediate which was quickly converted to **3a7** by the intramolecular proton transfer, and the glycosidic C–C bond cleavage of **2a2C** completed as **2a2C** → **3a8** + **C** → **3a7** + **C**. Accordingly, the reaction coordinate including two transition state complexes was built as shown in Fig. 7. The proposed two-step reaction mechanism was comprised of endergonic first reaction step and highly exergonic second reaction step.

The first activation energy of 37.28 kcal/mol seems to be a little high, but two factors can be considered for the plausibility of the proposed reaction pathway. First, the entropic contributions of the cleavage reaction should be included for the Gibbs free energy of activation. Though it is not straightforward, the structural changes observed from the reaction step **2a2C** to **TS1** (Fig. 7) suggests a significant increase in entropy^28^. Second, it has be kept in mind that the proposed reaction pathway did not consider the local environments of the enzyme active site. Enzyme can reduce the activation energy of the reaction by several mechanisms; proximity and orientation effects, general acid-general base catalysis, and especially stabilization of the transition state complex^29^. Usually, combined effects of several mechanism are employed to reduce the activation energy of the enzyme-catalyzed reaction, resulting in the rate enhancements of 10^10^–10^23^^30,31^. If the Eyring equation was applied for the values, 13–31 kcal/mol of decrease in Gibbs energy of activation is expected. Therefore, the activation energy of the proposed reaction pathway can be accessible in the enzyme-catalyzed C–C bond cleavage reaction.

**Figure 8:**
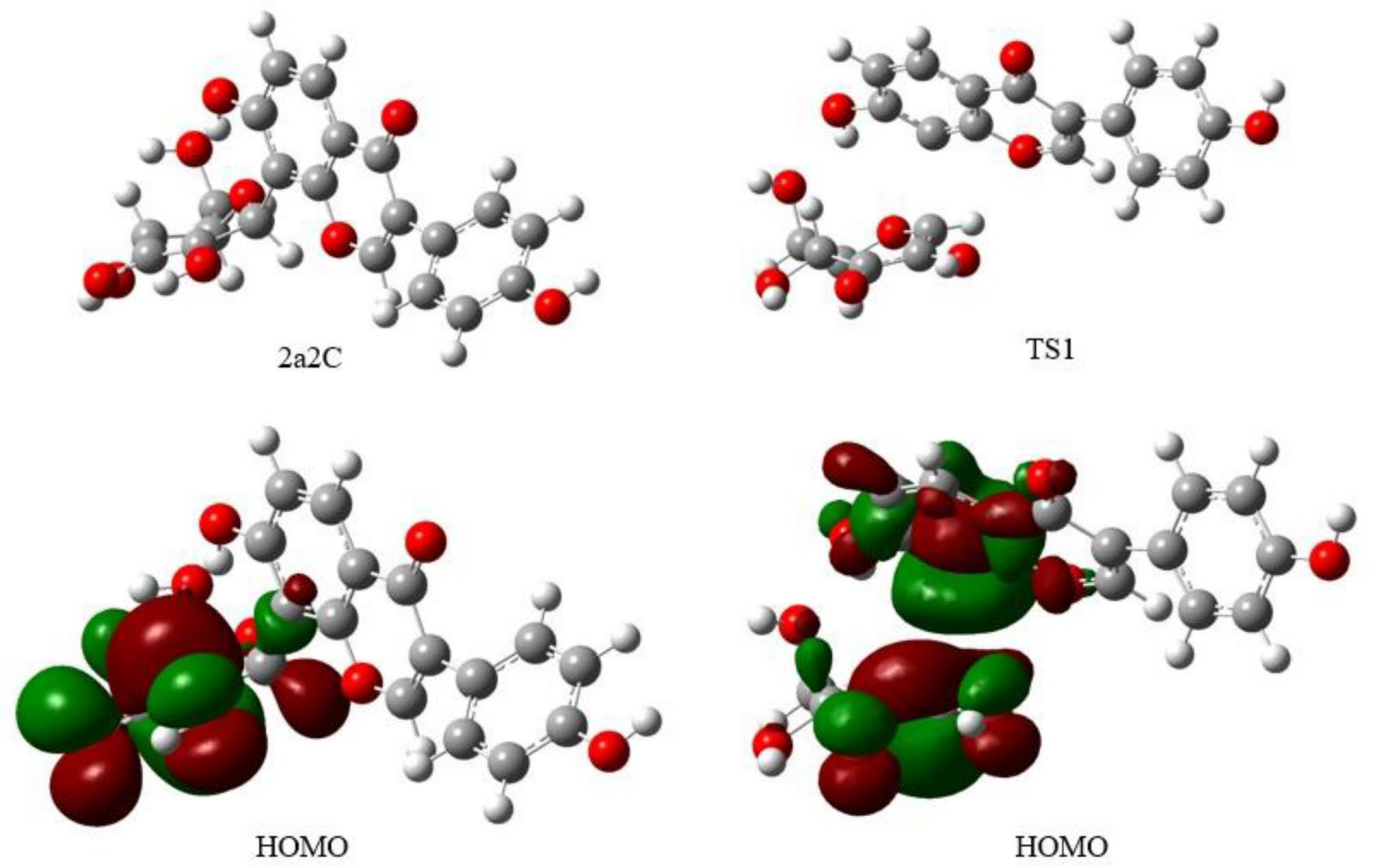
Molecular structures and orbitals (HOMO) of 2’’C-dehydro-3’’-oxo-puerarin (**2a2C**) and transition state complex 1 (**TS1**). HOMO of **2a2C** has σ-character bonding orbital, while HOMO of the first TS complex has π-antibonding orbital at the C8–C1’’ bond.

One of the characteristics of the proposed reaction mechanism is the significant conformational change of the glycosidic unit. The conformation of the pyranosyl ring changed from perpendicular to parallel to the isoflavone ring during the reaction. In Fig. 8, it is clear that HOMO of **2a2C** has σ-character bonding orbital, while HOMO of the first TS complex has π-antibonding orbital at the C8–C1’’ bond. The major difference between our proposed mechanism and previous one is the initiation of the reaction, deprotonation of 2’’C–H proton vs. quinoid formation.

In this report, theoretical study on the puerarin metabolism and the *C*-glycosidic bond cleavage were performed by DFT calculations using the B3LYP exchange correlation functional at the 6‐311++ G(d,p) basis set level. Biochemical energetics of puerarin conversion to daidzein and glucose was found to be slightly endergonic by 4.99 kcal/mol. It was also found that the quinoid intermediate (**4**), previously suggested as the key reaction intermediate, was thermodynamically disfavored due to the high energy barrier. From our computational study, it was newly proposed that the C–C bond cleavage reaction should be initiated by the deprotonation of 2’’C–H, and the carbanion intermediate (**2a2C**) performs C8–C1’’ bond cleavage reaction to produce hexose enediolone (**C**) and 8-dehydro-daidzein (**3a8**) products. The latter is quickly protonated to daidzein (**3**) through the daidzein anion (**3a7**).

## Computational methods

Molecules were built by GaussView 6, and geometry optimization and energy were calculated by using the *Gaussian 16* package^32^. The stability of each reaction species was compared by using free energy obtained by DFT B3LYP/6–311++ G(d,p) calculations in *n*-octanol and ethanol, respectively. Because of two different medium environments at the substrate binding site and solution, the results obtained in *n*-octanol, and ethanol were discussed for the study related to the reaction mechanism and bioenergetics of puerarin metabolism, respectively.

Glucosyl substituent of puerarin (**1**) and other glycosides shown in Fig. 1 have freedom of rotation at the B-ring and glucopyranosyl group. The rotational energy barriers of puerarin (**1**) and 3’’-oxo-puerarin (**2**) were calculated with the relaxed scan option from the geometry-optimized structures, by using the ModRedudant menu. Boltzmann distributions of the conformers were calculated after obtaining the Gibbs free energy from DFT calculation. For the Gibbs free energy comparison, the structures of all stationary points were fully optimized at the B3LYP level with 6–311++ G(d,p) basis sets. Grimme’s empirical dispersion with the D3 damping was included for all calculations^33^. The solvent effect was also included to model the environment by using the Solvation Model based on the quantum mechanical charge Density of a solute molecule interacting with a continuum^34^. Solution-phase vibrational frequencies were computed for each stationary points, and the minima and transition states (TSs) were confirmed to have zero and one imaginary frequency, respectively. For the Gibbs free energy in solution, a standard state correction of + 1.894 kcal/mol (0.00302 Hartree) at 298 K was included to transfer from an ideal gas of 1 atm to an ideal solution at a liquid-phase concentration of 1 mol/L^35^. The vibrational frequencies lower than 100 cm^−1^ were increased to 100 cm^−1^ to correct the breakdown of the harmonic oscillator model for the free energies of low-frequency vibrational mode^36^.

### Supplementary Information

Supplementary Table S1.

## Data Availability

All data generated or analyzed during this study are included in this published article. All materials are stored at the Computing Lab of College of Natural Resources and Biotechnology, Chung-Ang University Anseong, South Korea. The datasets used during the current study available from the corresponding author on every request.
