# Supplementary material for: Theoretical study on the glycosidic C–C bond cleavage of 3’’-oxo-puerarin
Source: Sci Rep. 2023 Sep 28;13:16282. doi: 10.1038/s41598-023-43379-1 (PMC10539306; doi:10.1038/s41598-023-43379-1)
Supplement: Supplementary file 1 — Supplementary Table S1. [file 41598_2023_43379_MOESM1_ESM.docx]

**Supplementary Information to**

**Theoretical study on the glycosidic C-C bond cleavage of 3”-oxo-puerarin**

Jongkeun Choi^1^, Yongho Kim^2^, Bekir Engin Eser^3^ & Jaehong Han^4^*

**Table S1.** Free energy (Hartree) of the compounds calculated by DFT B3LYP/6-311++g(d,p) in different solvents. The free energies were corrected by upscaling lower frequencies to 100 cm^-1^ by KiSThelP software and adding a standard state correction of +1.894 kcal/mol (0.00302 Hartree) at 298 K.

| **Compound** | ***n*-Octanol** | **Ethanol** |
| --- | --- | --- |
| puerarin (**1**) | -1489.46664 | -1489.47537 |
| 3”-oxo-puerarin (**2**) | -1488.27476 | -1488.28185 |
| 3”-oxo-puerarin anion (**2a7O**) | -1487.80920 | -1487.82254 |
| 2”C-dehydro-3”-oxo-puerarin (**2a2C**) | -1487.78981 | -1487.80145 |
| daidzein (**3**) | -878.62427 | -878.62884 |
| 8-dehydro-daidzein (**3a8**) | -878.10263 | -878.11186 |
| daidzein anion (**3a7**) | -878.16269 | -878.17172 |
| (8*R*)-3”-oxo-puerarin quinoid (**4*R***) | -1488.24748 | -1488.25580 |
| (8*S*)-3”-oxo-puerarin quinoid (**4*S***) | -1488.25039 | -1488.25869 |
| 2”C-dehydro-(8*R*)-3”-oxo-puerarin quinoid (**4*R*a2C**) | -1487.81059 | -1487.82194 |
| 2”C-dehydro-(8*S*)-3”-oxo-puerarin quinoid (**4*S*a2C**) | -1487.77138 | -1487.80920 |
| 3-oxo-glucose (**A**) | -686.10632 | -686.11069 |
| β-glucose (**B**) | -687.29924 | -687.30392 |
| hexose enediolone (**C**) | -609.64947 | -609.65266 |
| water | -76.46374 | -76.46534 |
| TS1 | -1487.73359 | nc |
| **C** + **3a8** complex | -1487.74478 | nc |
| TS2 | -1487.71521 | nc |
| **C** + **3a7** complex | -1487.80797 | nc |

* Energy was not calculated.
